# An integrated general practice and pharmacy-based intervention to promote the use of appropriate preventive medications among individuals at high cardiovascular disease risk: protocol for a cluster randomized controlled trial

**DOI:** 10.1186/s13012-016-0488-1

**Published:** 2016-09-23

**Authors:** Adina Hayek, Rohina Joshi, Tim Usherwood, Ruth Webster, Baldeep Kaur, Bandana Saini, Carol Armour, Ines Krass, Tracey-Lea Laba, Christopher Reid, Louise Shiel, Charlotte Hespe, Fred Hersch, Stephen Jan, Serigne Lo, David Peiris, Anthony Rodgers, Anushka Patel

**Affiliations:** 1 The George Institute for Global Health, Sydney, New South Wales Australia; 2 University of Sydney, Sydney, New South Wales Australia; 3 Sydney Medical School Westmead, University of Sydney, Sydney, New South Wales Australia; 4 Woolcock Institute, Sydney, New South Wales Australia; 5 School of Pharmacy, University of Sydney, Sydney, New South Wales Australia; 6 Monash University, Melbourne, Victoria Australia; 7 Curtin University, Bentley, Western Australia Australia; 8 School of Public Health and Preventive Medicine, Monash University, Melbourne, Victoria Australia; 9 University of Notre Dame, Sydney, New South Wales Australia; 10 Menzies Centre for Health Policy, School of Public Health, Sydney Medical School, The University of Sydney, Sydney, Australia

**Keywords:** Cardiovascular disease, General practitioners, Pharmacists, Clinical decision support system, Polypill, Primary health care

## Abstract

**Background:**

Cardiovascular diseases (CVD) are responsible for significant morbidity, premature mortality, and economic burden. Despite established evidence that supports the use of preventive medications among patients at high CVD risk, treatment gaps remain. Building on prior evidence and a theoretical framework, a complex intervention has been designed to address these gaps among high-risk, under-treated patients in the Australian primary care setting. This intervention comprises a general practice quality improvement tool incorporating clinical decision support and audit/feedback capabilities; availability of a range of CVD polypills (fixed-dose combinations of two blood pressure lowering agents, a statin ± aspirin) for prescription when appropriate; and access to a pharmacy-based program to support long-term medication adherence and lifestyle modification.

**Methods:**

Following a systematic development process, the intervention will be evaluated in a pragmatic cluster randomized controlled trial including 70 general practices for a median period of 18 months. The 35 general practices in the intervention group will work with a nominated partner pharmacy, whereas those in the control group will provide usual care without access to the intervention tools. The primary outcome is the proportion of patients at high CVD risk who were inadequately treated at baseline who achieve target blood pressure (BP) and low-density lipoprotein cholesterol (LDL-C) levels at the study end. The outcomes will be analyzed using data from electronic medical records, utilizing a validated extraction tool. Detailed process and economic evaluations will also be performed.

**Discussion:**

The study intends to establish evidence about an intervention that combines technological innovation with team collaboration between patients, pharmacists, and general practitioners (GPs) for CVD prevention.

**Trial registration:**

Australian New Zealand Clinical Trials Registry ACTRN12616000233426

## Background

Cardiovascular disease (CVD) causes significant health burden globally. Like many other high income nations, CVD is the leading cause of death in Australia, accounting for 31 % of all deaths [1]. CVD is also responsible for significant morbidity and economic burden, emphasizing the importance of effective prevention in primary care.

Numerous robust, large-scale clinical trials have demonstrated the benefits and safety of pharmacotherapy for CVD risk reduction. In high-risk patients, blood pressure lowering [2], lipid lowering [3], and antiplatelet therapies (in secondary prevention) [4, 5] have all been shown to reduce the risks of CVD events. However, appropriate prescribing is demonstrably suboptimal [6, 7], with a range of patient and health system factors adversely influencing long-term medication adherence [8]. A Cochrane review concluded that complex interventions which provide education, counselling, or daily treatment support were likely to be most effective in promoting long-term medication adherence [9].

Previous work by The George Institute for Global Health has developed a multifaceted quality improvement intervention (*HealthTracker*) comprising point-of-care computerized decision support, audit tools, and staff training for use in the general practice setting. *HealthTracker* has been described in detail elsewhere [10], but a key component is an algorithm that extracts data from the electronic medical record, using absolute CVD risk estimation and the recommendations of relevant guidelines to provide individualized advice on CVD risk factor measurement and treatment. Evaluation in a cluster randomized trial demonstrated that *HealthTracker* was associated with increased CVD risk assessment and escalation of medical therapy, with a 60 % relative improvement in use of optimal combination therapy among under-treated high-risk individuals [11]. Despite these effects, substantial treatment gaps persisted.

In parallel, a range of trials have evaluated the role of cardiovascular “polypills” (fixed-dose combinations of blood pressure lowering drugs and statins, with or without antiplatelet drugs) in improving adherence to optimal preventive drug therapy among individuals at high CVD risk. It had been posited that both prescription of appropriate therapy and adherence to prescribed medication would be enhanced with the availability of such polypills because of reduced costs and simplification of complex treatment regimens. A recent meta-analysis of three randomized controlled trials (including one in Australian primary care) indicated that a polypill-based strategy significantly improved medication adherence as well as reduced systolic blood pressure (BP) and low-density lipoprotein cholesterol (LDL-C) levels [12]. However, in all three studies, a decline in medication adherence over the 12–18-month period of observation was observed.

In many healthcare systems, including that of Australia, pharmacists are well placed to be involved in patient care to maintain long-term medication adherence. Patients with chronic diseases visit their pharmacists on average twice as frequently as their general practitioners (GPs) [13] for repeat medications, and such visits may represent an ideal opportunity to support patients in maintaining adherence. Existing data support the potential effectiveness of pharmacy-based interventions, with evidence of improved medication adherence with fee-for-service medication reviews [14], education/counselling [15–19], and team collaboration [15, 18, 20, 21] models. These interventions have been implemented in the management of hypertension [16, 17, 20–23], type 2 diabetes mellitus [19], and asthma [18].

This study aims to assess whether combining (a) *HealthTracker* with (b) the availability of a range of cardiovascular polypills and (c) a pharmacy-based adherence program will improve CVD preventive medication prescribing and adherence in patients at high absolute CVD risk. Utilizing an appropriate framework to theorize the interaction of these combined components, a complex intervention is being proposed. This paper describes development of the intervention and pragmatic cluster randomized trial that will evaluate its clinical and cost-effectiveness, and potential scalability and sustainability beyond the trial setting.

## Methods/design

### Description of intervention

We have used Michie’s behavior change model [24] and process evaluations of our previous work [25–27] to develop an understanding of the capabilities, opportunities, and motivations of patients and their health providers, as well as to theorize the expected contribution of each component and their interaction (e.g., polypill increases opportunity by reducing cost and tablet burden; decision support tools increase patient motivation and practitioner capability, and individualized pharmacy support [18] increases patient motivation). This theory-driven approach, informed by our empirical findings, provides a strong rationale for the multi-component intervention and a reasonable expectation that the individual components may potentiate one another to yield larger effect sizes.

**Fig. 1:**
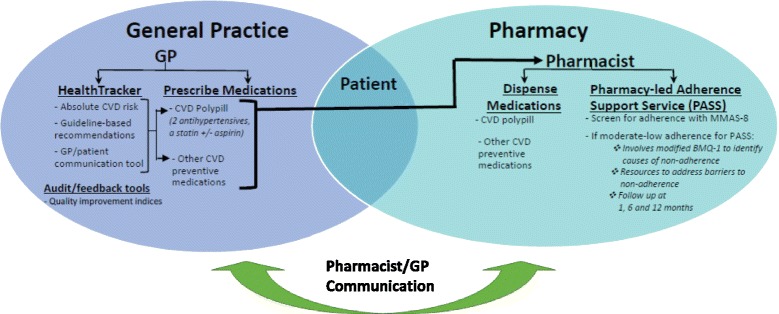
INTEGRATE intervention

The intervention will involve GPs, pharmacists, and patients (Fig. 1). GPs will have *HealthTracker* uploaded on their clinical computer, linked to a server-based data extraction tool [28] both of which integrate with their electronic medical records (EMRs). Utilizing data within the EMR, *HealthTracker* automatically calculates the absolute CVD risk of a patient whose record is open or alerts the GP if outstanding risk factors need to be measured in order to estimate risk [10]. A risk communication tool allows GPs to interact with patients about their CVD risk and potential for altering their risk based on beneficial or harmful changes to risk factor levels [29]. It recommends patient-appropriate lifestyle changes with pertinent patient resources (e.g., weight loss information sheets, quit smoking phone apps) and prescription of medications (including the option, where relevant, of cardiovascular polypills) based on the simultaneous interpretation of multiple clinical guidelines and given the known characteristics of the patient. The data extraction tool will provide GPs with collated data on their clinical performance for key indicators and will include the capacity to compare performance with other de-identified peer practices.

*HealthTracker* will also provide tailored advice in relation to eight variations of a CVD polypill that are available for this study (Table 1) through the process of over-encapsulation. Over-encapsulation is a method of securely enclosing solid dosage forms of medications inside a capsule shell. Polypill prescriptions will be able to be filled only at partner pharmacies. Out-of-pocket expenses for the polypill will be incurred by participating patients identically to those for any other drug listed on the Pharmaceutical Benefits Scheme (PBS), which is the government subsidy program through which most drugs are obtained in Australia [30]. This cost varies depending on whether or not the patient is eligible for any concessions. Since January 2016, the patient co-payments for non-concession and concession card holders are $AUD38.30 and $AUD6.20, respectively [31]. The equivalent co-payment for one medication will be charged for any polypill prescription to replicate clinical practice as closely as possible, and the participating pharmacy will retain this fee as the cost for their dispensing services. However, CVD polypills are not currently available on the Australian market and will not contribute towards patient safety net entitlements [32].

**Table 1:** CVD polypills for use in the INTEGRATE study

Tablet name	ACEI/ARB	Second antihypertensive agent	Statin	Antiplatelet agent
“Polypill Perindap Asp”	Perindopril erbumine (4 mg)	Indapamide (1.25 mg)	Rosuvastatin (10 mg)	Aspirin (100 mg)
“Polypill Perindap”	Perindopril erbumine (4 mg)	Indapamide (1.25 mg)	Rosuvastatin (10 mg)	–
“Polypill Hydrotelmi Asp”	Telmisartan (40 mg)	Hydrochlorothiazide (12.5 mg)	Rosuvastatin (10 mg)	Aspirin (100 mg)
“Polypill Hydrotelmi”	Telmisartan (40 mg)	Hydrochlorothiazide (12.5 mg)	Rosuvastatin (10 mg)	–
“Polypill Peramlo Asp”	Perindopril erbumine (4 mg)	Amlodipine (5 mg)	Rosuvastatin (10 mg)	Aspirin (100 mg)
“Polypill Peramlo”	Perindopril erbumine (4 mg)	Amlodipine (5 mg)	Rosuvastatin (10 mg)	–
“Polypill Amtelmi Asp”	Telmisartan (40 mg)	Amlodipine (5 mg)	Rosuvastatin (10 mg)	Aspirin (100 mg)
“Polypill Amtelmi”	Telmisartan (40 mg)	Amlodipine (5 mg)	Rosuvastatin (10 mg)	–

*ACEI* angiotensin converting enzyme inhibitor, *ARB* angiotensin receptor blocker

Patients with any prescriptions for preventive cardiovascular medications (including but not limited to a CVD polypill) will be eligible for referral by the GP to a partner pharmacy for potential involvement in the Pharmacy Adherence Support Service (PASS). The pharmacist will undertake initial screening with the eight-item Morisky Medication Adherence Scale (MMAS-8) [33–35] to identify patients at moderate or high-risk of medication non-adherence. With the aid of an electronic decision support system, pharmacists will then use a modified Brief Medication Questionnaire-1 (BMQ-1) to assess reasons for non-adherence [36] and address these barriers. Follow-up PASS assessments and interventions will occur at 1, 6, and 12 months following initiation. Pharmacists will be remunerated for their time spent with patient who participates in this program, commensurate with a fee structure for other services that currently generate reimbursement.

A secure electronic communication platform between the pharmacists’ decision support tool (PASS program) and GPs’ health records will be established to facilitate communication between GPs and pharmacists. This will facilitate pharmacists to send automated letters to GPs directly from the PASS application to detail patient progress, as well as provide an opportunity for other individualized interdisciplinary communication.

### Pilot phase

Prior to large-scale implementation and evaluation of the intervention, a pilot will be completed in at least three general practice and pharmacy pairs for up to 8 weeks (including a minimum of 4 weeks of follow-up after the last patient has been included in the PASS program). Clinical data and measures of fidelity will be collected (for example, frequency of *HealthTracker* utilization). In-depth interviews involving GPs, pharmacists, and a sample of patients will identify barriers to the implementation of the intervention to be addressed prior to wider application. The pilot study will provide an initial understanding of the operation of the study, including implementation, mechanism of impact, and context for overall process evaluation [37].

### Trial design

**Fig. 2:**
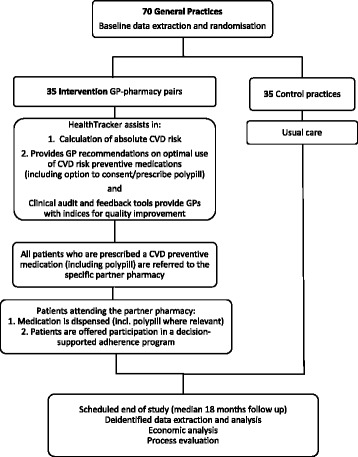
INTEGRATE study outline

The INTEGRATE study is planned as an open label, pragmatic, cluster randomized controlled trial of 70 general practices in Australia (Fig. 2).

#### Study population

General practices with EMR systems compliant with *HealthTracker* use will be eligible (currently estimated as at least 80 % of GP practices in Australia). Practices with previous exposure to *HealthTracker* will be excluded. The patient population for evaluation is based on Australian vascular risk screening recommendations [38] and defined as all Aboriginal and Torres Strait Islander people ≥35 years and all others ≥45 years (no upper age limit) who had attended the service ≥3 times in the previous 24-month period and at least once in the previous 6-month period. The outcome evaluation cohort will include patients who met these criteria at both baseline and end of study and will be de-identified prior to data extractions from each practice; however, an encrypted identifier will be utilized to enable matching of the data.

#### Randomization

Permuted block randomization will be centrally computer generated and stratified by general practice size (<500 patients vs ≥500 patients) and location (metropolitan vs non-metropolitan by rural, remote, and metropolitan Area (RRMA) classification [39]).

#### Post-randomization phase

Those practices randomized to the intervention will have *HealthTracker* installed in all clinical computers, and the staff will be provided training in its use. Each practice will be paired with a partner pharmacy that uses electronic dispensing software and is willing to dispense the polypill and deliver the PASS program. Brief education will also be provided to the GP about the availability and use of polypill therapy and to the pharmacist in delivery of the PASS program. Pharmacies will be assisted in establishing systems to stock and re-order supplies of the polypills. Once the training is delivered and the intervention is commenced, there will be minimal involvement of study staff, other than through a Help Desk support function. The general practices assigned to usual care will not be paired to partner pharmacies nor have access to *HealthTracker*, polypill prescribing or PASS program referrals.

#### Outcomes

The primary effectiveness outcome is the proportion of patients at high CVD risk who were not on full preventive treatment at baseline (“under-treated”) who achieve recommended target BP and LDL-C levels at study end. High CVD risk is defined by current Australian guidelines as those with a history of established CVD, presence of high-risk conditions (diabetes and age over 60 years, diabetes and albuminuria, stage 3B chronic kidney disease, systolic BP ≥180 mmHg, diastolic BP ≥110 mmHg, or total cholesterol >7.5 mmol/L), or a calculated 5-year CVD risk of >15 % using the 1991 Anderson Framingham equation [38]. Full preventive treatment is defined as a combination of antiplatelet drug, at least one blood pressure lowering medication and a statin in those with established atherothrombotic CVD, and the combination of at least one blood pressure lowering medication and a statin in all other high-risk patients. Target levels are defined as BP ≤140/90 mmHg or ≤130/80 mmHg in people with diabetes or albuminuria, and LDL-C <2.0 mmol/L.

Secondary effectiveness outcomes include the proportion of under-treated high CVD risk patients at baseline achieving recommended target BP at end of study; the proportion of under-treated high CVD risk patients at baseline achieving recommended target LDL-C at end of study; the proportion of all high CVD risk patients achieving BP and LDL-C targets at end of study; the proportion of patients achieving BP and LDL-C targets and prescribed antiplatelet agent (if relevant) at end of study; recording of risk factor measurement and mean levels during the study period; treatment intensification in high risk-patients; and the proportions of non-high CVD risk patients receiving BP lowering, statin, and antiplatelet therapy at the end of the study.

Seventy general practices with a mean patient cluster size of 60 will provide at least 80 % power (2*α* = 0.05) to detect a relative risk of ≥1.35 in the proportion of high CVD risk patients achieving BP and LDL-C targets between the intervention and control arms. This is based on the assumptions of an intraclass correlation coefficient of 0.01 (from a previous trial of *HealthTracker*) and that 10 % of relevant patients in the usual care arm will have BP and LDL-C levels at or below target by the study end.

Individual patient data will be analyzed on an intention-to-treat basis using Gaussian and log-binomial generalized estimating equation regressions for continuous and binary outcomes, respectively, and will account for clustering of patients within practices. Pre-specified subgroup analyses will also be performed using the randomization strata.

#### Economic and process evaluation

An economic evaluation of INTEGRATE will be conducted to estimate the incremental costs and benefits of the intervention to the health system. This evaluation will comprise a trial-based component as well as a modelled evaluation of the long-term costs and outcomes. Cost estimates will be based on all aspects of the intervention, including direct medical utilization costs (e.g., general practitioner consultations, pathology obtained from the data extraction tools and valued at prevailing costs) and intervention costs (e.g., training, tablet devices, software, and IT support, practice implementation costs). The incremental cost-effectiveness of the intervention in achieving each of the study outcomes will then be estimated. To understand the cost-effectiveness of INTEGRATE beyond the trial setting, a Markov model will be used to predict the long-term intervention effects and costs. The model will be based on tracking the progression of a cohort of surviving patients at the end of the trial over decades in which they potentially progress, based on annual cycles, through a number of health states including no disease, death, myocardial infarction, and stroke. Patients in the intervention group will be tracked separately from those in the usual care group. We will draw on published evidence to determine the transition probabilities between health states, costs associated with CVD events, and quality of life. Incremental cost-effectiveness ratio will be estimated by folding back the model at a point in time in the future in which patients in both groups have all died and the difference in accumulated quality-adjusted life-years (QALYs) and costs will be estimated (discounted at an appropriate rate). These analyses will provide information on the investment case for INTEGRATE, with appropriate sensitivity analysis to account for variations that may occur across different settings and thus address issues of generalizability and scalability.

**Fig. 3:**
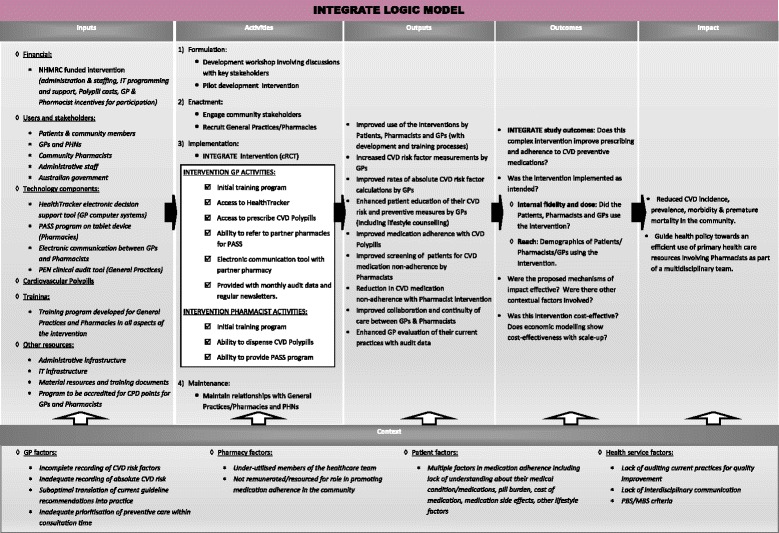
INTEGRATE logic model

A detailed process evaluation will be crucial in the interpretation of the effectiveness findings. This will assess whether INTEGRATE was delivered as planned (fidelity, dose, and reach), the mechanisms by which the study outcomes were achieved (or not achieved) and how contextual factors impacted on the findings [37]. A logic model [40] (Fig. 3) will be used to evaluate the feasibility of upscaling and the potential application of INTEGRATE to different “real life” settings. The process evaluation will involve mixed methods evaluation including quantitative data collection on the usage of the different components of the study and semi-structured interviews with patients, pharmacists, and GPs. The interviews will provide information on the experiences and perspectives of patients and health care providers with purposive sampling to ensure diverse opinions are gained from groups with high and low uptake of the intervention and will continue until thematic saturation is achieved.

### Ethical considerations

INTEGRATE has been approved by the University of Sydney Human Research Ethics Committee (HREC) and ratified by the corresponding ethics committees at Monash University and the University of Notre Dame. Only de-identified patient data will be collected for the purpose of the study and will be securely stored at The George Institute. Written informed consent will be obtained from all patients who are prescribed the polypill or who participate in the PASS program, as well as from all patients, pharmacists, and GPs who participate in the interviews for the process evaluation.

### Timelines

The pilot development phase will be implemented from the second quarter of 2016. INTEGRATE will subsequently begin with pre-randomization baseline data extraction followed by trial implementation in the third quarter of 2016 and will conclude in the first quarter of 2019, followed by data analysis and dissemination of results.

## Discussion

Many CVD deaths are preventable with lifestyle measures and medication management. Yet, despite efficacious treatments being available, significant morbidity and premature mortality persist; therefore, this study aims to trial a systems approach to the ongoing burden of CVD. As well as population-based approaches, attention needs to be focused on the large numbers of high-risk, under-treated patient populations for maximum public health benefit. The primary care setting is ideal for these preventative interventions due to the high frequency of patient encounters, with 83 % of Australians attending a GP every year [41].

The INTEGRATE intervention aims to simultaneously address a number of steps in health professional and patient interactions that may lead to suboptimal care or provide opportunities for improved care. These steps include risk factor measurements (such as BP, LDL-C), estimation of absolute CVD risk, risk communication, appropriate treatment recommendations (including lifestyle counselling) and prescriptions, patient education, addressing barriers to adherence, and facilitating interdisciplinary communication between GPs and pharmacists. While there is growing evidence that a variety of isolated interventions such as electronic decision support can aid this process, INTEGRATE aims to provide evidence of the potentially synergistic effectiveness of a model of care combining technological innovation with team collaboration between pharmacists and GPs. In the Australian context, this is consistent with the new “medical home” model of patient-centered care involving continuity of care with interdisciplinary collaboration, as well as the need for ongoing quality improvement to enhance systems of healthcare delivery [42].

Some potential limitation should be acknowledged. Polypills are not available for prescription in Australia, and despite using the process of over-encapsulation of marketed generic drugs, these are considered unapproved therapeutic agents from a regulatory perspective, and written informed consent is required from patients for prescription of polypills as part of this study. The INTEGRATE intervention has been carefully designed to minimize disruption to workflow for participating GPs in particular; however, this required trial process has the potential to significantly undermine this principle. Consequently, polypill use in the trial may not reflect likely use in “real world” practice and may lead to under-estimation of the overall effectiveness of the overall intervention. While unlikely in the context of a cluster randomized trial with geographically dispersed practice-pharmacy pairs, it is possible that a control group patient may attend a pharmacy in the intervention group. Training of pharmacists will include reminding pharmacists to restrict the PASS program to patients from GP practices randomized to the intervention.

The ongoing process of refining the INTEGRATE intervention will be highly influenced by local context. While the process evaluation will attempt to define features of the context that might help explain who it works best for and under which conditions, this will almost certainly limit direct extrapolation of the effectiveness findings from the trial to other settings with different health care systems. Nevertheless, efforts to understand the effects of “function over form” through the process evaluation may help inform potential adaption to other health systems.

## Abbreviations

ACEI: Angiotensin converting enzyme inhibitor
ARB: Angiotensin receptor blocker
BMQ-1: Brief medication questionnaire-1
BP: Blood pressure
CVD: Cardiovascular disease
EMR: Electronic medical record
HREC: Human Research Ethics Committee
LDL-C: Low-density lipoprotein cholesterol
MMAS-8: Morisky medication adherence scale-8
PASS: Pharmacy-led adherence support service
PBS: Pharmaceutical Benefits Scheme
QALYs: Quality adjusted life-years
